# IgM-Linked SerpinB3 and SerpinB4 in Sera of Patients with Chronic Liver Disease

**DOI:** 10.1371/journal.pone.0040658

**Published:** 2012-07-13

**Authors:** Alessandra Biasiolo, Natascia Tono, Mariagrazia Ruvoletto, Santina Quarta, Cristian Turato, Gianmarco Villano, Luca Beneduce, Giorgio Fassina, Carlo Merkel, Angelo Gatta, Patrizia Pontisso

**Affiliations:** 1 Department of Medicine, University of Padova, Padova, Italy; 2 Istituto Oncologico Veneto (IOV), IRCCS, Padova, Italy; 3 Xeptagen S.p.A., Venice, Italy; Klinikum rechts der Isar der TU München, Germany

## Abstract

**Background:**

Epidemiological studies indicate that a growing number of cirrhotic patients will develop hepatocellular carcinoma (HCC) in the next decade. Recent findings have demonstrated that Squamous cell carcinoma antigen 1 (SCCA1) and 2 (SCCA2) isoforms, now classified as serpinB3 and serpinB4, are over-expressed in HCC, but not in normal liver. As reported, high levels of circulating SCCA-IgM immunocomplexes in patients with cirrhosis are significantly associated with HCC development.

**Aim:**

To ascertain whether IgM-linked SCCA isoforms circulate in patients with chronic liver disease, compared to total SCCA-IgM levels.

**Methodology and Findings:**

79 patients with chronic liver disease were studied, including 17 patients with chronic hepatitis, 36 patients with cirrhosis and 26 with HCC. 28 blood donors were used as control. Monoclonal antibodies against serpinB3 and serpinB4 were used as catcher antibodies to set up specific ELISA assays, while total SCCA-IgM immunocomplexes were detected by commercially available ELISA assay. Overall, the results revealed a better diagnostic sensitivity of total SCCA-IgM assay, compared to both serpinB3 and serpinB4 IgM-linked assays. SerpinB4-IgM median values obtained with SCC103 antibody were moderately higher in patients with cirrhosis than in those with HCC, median values: 0.168 (IQR 0.140–0.427) vs. 0.140 (IQR 0.140–0.278), (p = 0.177). A trend toward decreasing serpinB4-IgM/serpinB3-IgM median ratio was observed in patients with advanced liver disease, being 1.08 in patients with HCC, 1.10 in patients with cirrhosis and 1.40 in patients with chronic hepatitis (p = 0.079).

**Conclusions:**

IgM-linked SCCA isoforms in serum of patients with chronic liver diseases were quantified for the first time. Although the number of patients was limited, this preliminary study reveals that the relative balance of the two serpin isoforms is altered in HCC and it is characterized by a lower serpinB4-IgM/serpinB3-IgM ratio, determined by lower serpinB4 levels.

## Introduction

SerpinB3 and serpinB4 isoforms, also known as squamous cell carcinoma antigen 1 and 2 (SCCA1 and SCCA2) belong to ov-serpin/clade B serpin family [1]. Over 1500 serpin members have been identified in humans, plants, bacteria, archea and poxviruses to date [2], [3].

Genomic cloning of these two isoforms revealed that they are highly homologous, 91% identical at the amino acid level [4], [5], share conserved tertiary structure, and use a unique conformational rearrangement for their inhibitory activity [6], [7]. However, serpinB3 and serpinB4 show distinct properties and substrates: serpinB3 is a papain-like cysteine proteinase inhibitor, while serpinB4 is a chymotrypsin-like serine proteinase inhibitor [8], [9]. Little is known concerning the regulation of their gene expression. Both isoforms are broadly co-expressed in the spinous and granular layers of normal squamous epithelium, in several organs including tongue, tonsil, oesophagus, uterine cervix, vagina, the conducting airways, Hassall’s corpuscles of the thymus and some areas of the skin [10]. Regarding their role in normal epithelia, it has been suggested that SCCA isoforms may protect from bacterial and viral cystein proteases [11], mast cell chymase [12] and may also prevent cellular apoptosis of the cornified layer.

It has been demonstrated that SCCA isoforms are often overexpressed in neoplastic cells of epithelial origin [13], although their biological role in cancer cell is still unclear. It has been reported that both serpinB3 and serpinB4 protect neoplastic cells from apoptosis [14] and that serpinB3 promotes tumour growth [5], [15]–[16], epithelial to mesenchymal transition and cell proliferation [17].

Overexpression of SCCA isoforms has been also described in HCC and in highly displastic liver nodules, but not in normal liver [18]–[20]. In addition, high levels of SCCA-IgM linked complexes, but not of the free SCCA protein, have been described in serum of patient with HCC [21].

To date, little information is available about the profile of expression of SCCA isoforms in patients with cancer. Some authors have demonstrated a selective expression of serpinB4 mRNA in squamous cell carcinoma (SCC) tissues from uterine cervix when compared to normal tissue or SCC tissues from oesophagus or skin [22]–[24]. Serological studies have reported elevated serum levels of serpinB4 isoform, ascribed to direct release from tumour cells [25], [26]. However, there is still conflicting information about the prevalent circulating SCCA isoform and additional studies have not confirmed these data [27].

According to the new theory about cancer immunosurveillance, now updated as immunoediting [28]–[30], natural IgMs seem to play an important role in the innate immune response, not only against infectious agents, but also in the immunosurveillance against tumour cell growth. Multivalent IgMs bear a characteristic capacity to bind a wide range of post-transcriptionally modified tumour antigens and they all induce cancer-specific apoptosis, by triggering the intrinsic apoptotic pathway [31]. Several studies have demonstrated that tumour released antigens can react with the natural IgM class of immunoglobulins and form circulating immune complexes in different human tumours. The described immunocomplexed antigens include CEA in colorectal cancer [32], PSA in prostate cancer [33], AFP, SCCA and DPC in liver cancer [21], [34], [35]. It has been also shown that these circulating immunocomplexes provide a better diagnostic performance than the corresponding free biomarker.

The purpose of this study was to evaluate the occurrence of serum immunoreactivity of IgM linked serpinB3 and serpinB4 isoforms in patients with different extent of chronic liver disease, compared to total SCCA-IgM levels.

## Results

The results obtained in the study, expressed as descriptive parameters, are summarized in Table 1. OD median value of serpinB3-IgM was 0.130 in each group of patients. The values of serpinB4-IgM obtained with SCC103 antibody, which recognises the serpin-protease complex, were slightly lower in patients with HCC, compared to patients with cirrhosis (median values: 0.140 (IQR 0.140–0.278) vs 0.168 (IQR 0.140–0.427 p = 0.177). Similar and not significantly different values of serpinB4-IgM obtained with SCC104 antibody were found comparing all groups of studied patients.

**Table 1:** IgM linked SerpinB3 and SerpinB4 isoforms, compared to total SCCA-IgM in patients with different extent of chronic liver disease.

		Chronic hepatitis	Cirrhosis	HCC	Healthy Subjects
		n = 17	n = 36	n = 26	n = 28
SerpinB3-IgM (SCC111)	Median (OD405 nm)	0.130	0.130	0.130	0.130
	IQR	0.130–0.172	0.130–0.221	0.130–0.175	0.130–0.130
	95^th^ percentile				0.200
	% positivity	12	28	23	7
SerpinB4-IgM (SCC103)	Median (OD405 nm)	0.207	0.168	0.140	0.154
	IQR	0.140–0.275	0.140–0.427	0.140–0.278	0.140–0.249
	95^th^ percentile				0.384
	% positivity	12	28	23	7
SerpinB4-IgM (SCC104)	Median (OD405 nm)	0.126	0.135	0.125	0.125
	IQR	0.125–0.227	0.125–0.285	0.125–0.319	0.125–0.125
	95^th^ percentile				0.199
	% positivity	29	42	35	7
Total SCCA-IgM	Median (AU/mL)	166	134	209	115
	IQR	80–511	80–552	118–287	109–129
	95^th^ percentile				156
	% positivity	53	47	58	0

IQR = interquartile range (25^th^–75^th^ percentile).

The percentage of positivity for each immunocomplex was determined as the fraction number of patients with immunocomplex levels above the defined cut-off value (95^th^ percentile).

SerpinB3-IgM complex was positive in 2 out of 17 (12%) patients with chronic hepatitis, in 10 out of 36 (28%) patients with cirrhosis and in 6 out of 26 (23%) patients with HCC. Similar values of reactivity were obtained for serpinB4-IgM detected immobilizing SCC103 antibody on the plate of the dedicated ELISA. A little gain in positivity rate for the detection of serpinB4-IgM in the three analyzed groups of patients was obtained using the other serpinB4 specific monoclonal antibody SCC104Ab. Using this latter antibody 5 out of 17 (29%) patients with chronic hepatitis, 15 out of 36 (42%) patients with cirrhosis and 9 out of 26 (35%) patients with HCC were positive. When total SCCA-IgM levels, regarded as the reference biomarker, were measured, 53% of the patients with chronic hepatitis, 47% of the cirrhotic patients and 58% of the patients with HCC were positive, showing a better sensitivity of this reference test, when compared with the sensitivity of the three above-listed assays. All healthy subjects were negative for total SCCA-IgM ELISA, while a 7% of positivity rate was found using the other ELISA assays.

**Figure 1:**
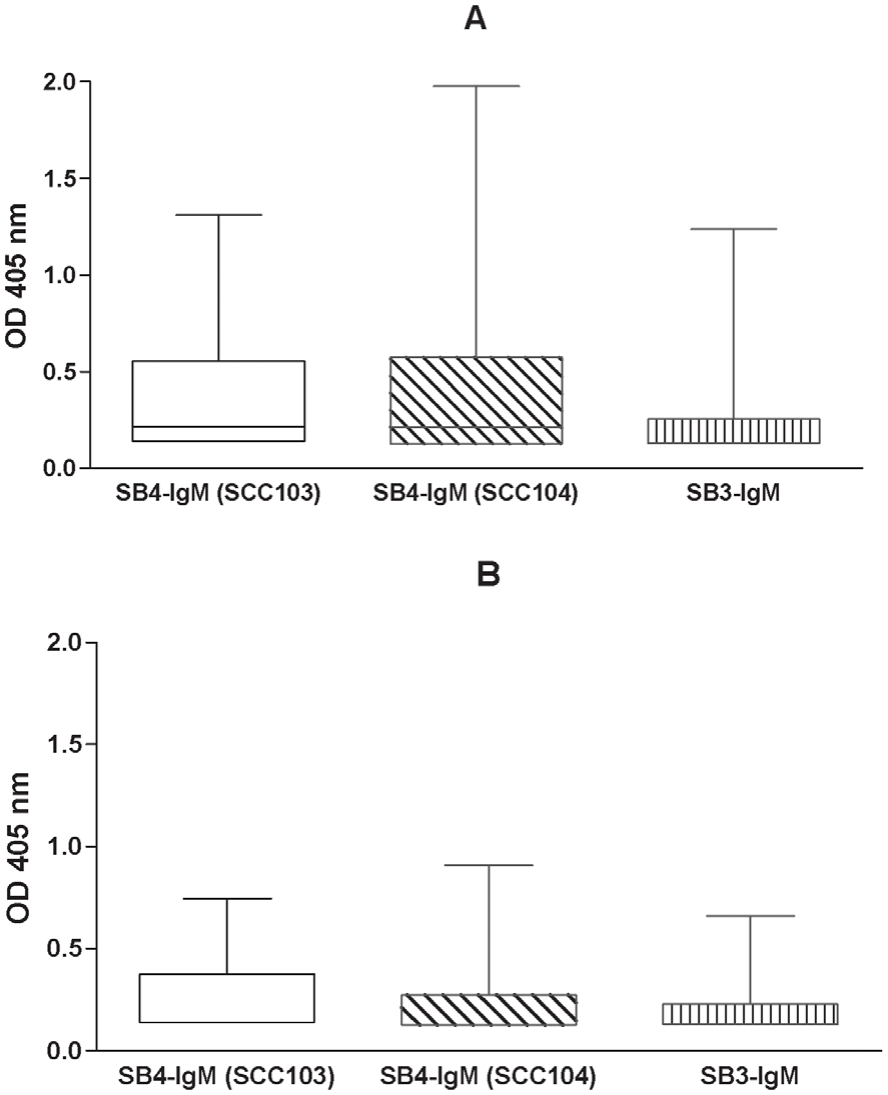
Serpin-IgM isoforms in cirrhotic patients with (panel A) and without (panel B) HCC evolution. Data in the box-plot graphs represent median, upper and lower OD values for serpinB4-IgM (SCC 103), serpinB4-IgM (SCC 104) and serpinB3-IgM.

Circulating levels of AFP were also measured, and were positive in 4 out of 26 (15%) patients with cirrhosis (median value 8.2 IU/ml) and in 13 out of 26 (50%) patients with HCC (median value 19.8 IU/ml; p = 0.02). In the group of patients with liver cirrhosis the distribution pattern of IgM-linked immunocomplexes was further analyzed in relation to liver tumour progression. Seventeen patients developed liver cancer during follow-up (group A) and these patients (Figure 1, panel A) showed higher, but not statistically significant, OD median values for all circulating biomarkers, when compared to patients without histological evolution (n = 19, group B) (Figure 1, panel B).

Several reports indicate that serpinB4, corresponding to the acidic isoform, is the main circulating isoform in patients with epithelial cancers [40], [41], [26] and an elevated serpinB4/serpinB3 mRNA ratio, detectable in different cancer cells [22], [23], [42], has been described as a poor prognostic factor for early-stage cervical cancer. In the present study, we have therefore evaluated the ratio between the different IgM-linked serpin isoforms in the three groups of patients.

**Figure 2:**
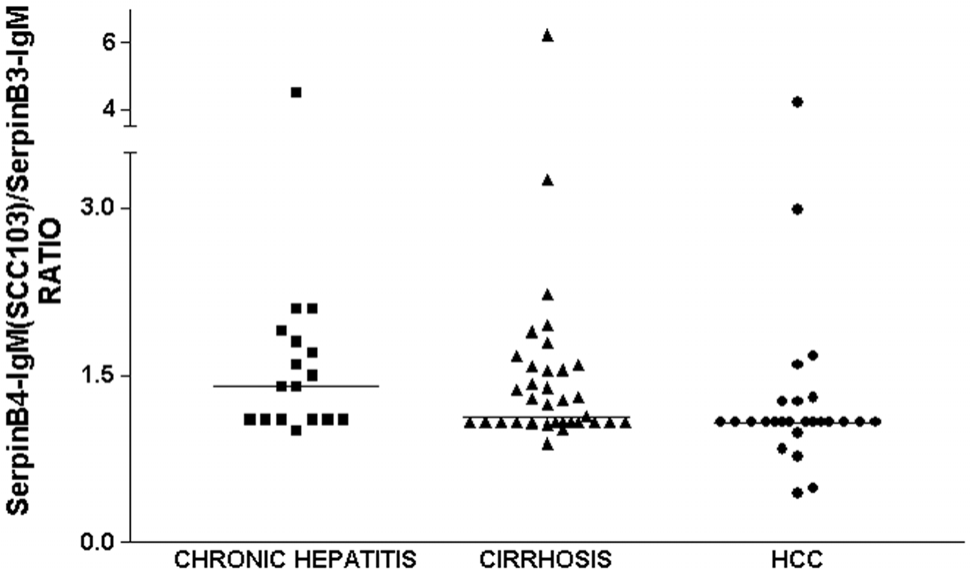
Distribution of serpinB4-IgM(SCC103)/serpinB3-IgM ratios. Scatter plot displays the distribution of serpinB4-IgM(SCC103)/serpinB3-IgM ratios detected in patients with chronic hepatitis, cirrhosis and HCC. Horizontal bars represent median value for each group.

In patients with chronic hepatitis the serpinB4-IgM(SCC103)/serpinB3-IgM median ratio was 1.40 (range 1.0–4.5), in patients with cirrhosis it was 1.10 (range 0.9–6.2), and in patients with HCC it was 1.08 (range 0.4–4.2). A progressive decrease of median ratio was observed in patients with more advanced liver disease, although no statistical difference was reached (Figure 2). These data are in keeping with the finding that 16 out of 26 patients with HCC (62%) and 15 out of 36 patients with cirrhosis (42%) were found below the LoD value for SerpinB4-IgM.

## Discussion

In the present study a simple assay to quantify the two isoforms serpinB3 and serpinB4 circulating as IgM-linked immunocomplexes in patients with different extent of chronic liver disease was set-up. The choice of analyzing the IgM-linked instead of the free isoforms was derived by the results obtained previously, when the assay to detect SCCA-IgM reactivity was initially described and performed better than that for the detection of free SCCA protein in serum [21]. Isoform-specific immunoenzymatic assays have been set up, using commercially available monoclonal antibodies. The aim of this investigation was to assess the pattern of expression of IgM-linked SCCA isoforms in chronic liver disease and to define whether they might provide any clinical advantage compared to total circulating SCCA-IgM levels.

To the best of our knowledge, this is the first study on the behaviour of circulating SCCA-IgM isoforms in patients with chronic liver diseases. We have recently shown a progressive increase over time of total SCCA-IgM immunocomplex in sera of untreated patients with progressive forms of chronic hepatitis [43] and in cirrhotic patients who developed liver cancer during follow-up [44]. Although the number of patients was limited, we have attempted to evaluate whether the reactivity for IgM-linked serpin isoforms was distributed differently in patients with chronic hepatitis and in cirrhotic patients with or without liver cancer progression. The small number of patients did not allow to identify significant differences between serpinB3-IgM and serpinB4-IgM behaviour, despite cirrhotic patients who developed HCC showed a trend to higher reactivity for all circulating biomarkers.

The majority of previous reports concerning SCCA levels in serum of patients with epithelial cancers describe serpinB4 as the dominant serological isoform [37], [40], [26]. These data were confirmed by histological studies, showing an elevated expression of serpinB4 in cancer tissues [22], [41], as well as an elevated serpinB4/serpinB3 mRNA ratio in cervical carcinoma [41], [23] and in head–neck cancer [45]. This effect was explained as a result of a possible protective role of serpinB4 from inflammation and apoptosis of tumour cells, probably due to direct inhibition of cathepsin G [23].

As for SCCA-IgM isoforms and liver cancer, our findings clearly document a graduate decrease of serpinB4-IgM/serpinB3-IgM ratio when comparing patients with different extent of liver disease, despite not reaching statistically significant differences due to the limited number of the patients included in the study. This trend was mainly a consequence of lower serum levels of serpinB4-IgM in patients with liver tumour, where the majority of them showed values below the detection limit for this assay. Despite our findings are not in agreement with published studies [37], [40], [26], it should be noted that current literature on this subject refers to the analysis of SCCA free isoforms in cancers of epithelial origin, where the corresponding normal tissues express physiologically this serpin [46], [10], while normal liver does not [18]. Since SerpinB3 has been found to induce epithelial to mesenchymal transition and increased proliferative and invasive potential (17), while no parallel information is available for SerpinB4, further studies to define the precise biological activities of the two isoforms, beside their antiprotease activity, are warranted.

In conclusion, the relative balance of the two serpin isoforms seems to be altered in HCC and characterized by a lower serpinB4-IgM/serpinB3-IgM ratio, determined by lower serpinB4 levels.

## Materials and Methods

### Patients

Serum samples from 28 blood donors and from 79 patients with different extent of liver disease, including 17 patients with histologically proven chronic hepatitis, 36 patients with histologically proven liver cirrhosis and 26 patients with HCC, were analyzed. None of the patients underwent antiviral treatment at least 12 months before serum sample collection. Patients with cirrhosis underwent regular liver ultrasound screening and were classified as Child A at the time of serum collection. The diagnosis of HCC was based on the presence of hepatic focal lesion >2 cm detected by liver ultrasound and confirmed by computed tomography and/or magnetic resonance as imaging techniques [36]. The final diagnosis was confirmed by histopathological analysis on ultrasound-assisted fine needle biopsy, when indicated.

In patients with cirrhosis, two groups were identified on the basis of HCC progression during follow-up: group A included 17 patients who developed HCC during a follow-up median period of 4 years, while the remaining 19 patients (group B) showed no histologic evidence of disease progression during the same time interval. Table 2 summarizes the main epidemiological and clinical characteristics of the study population: both groups of cirrhotic patients showed similar clinical profiles in terms of mean age, sex distribution and aetiology. Overall, patients were prevalently male, with mean age ranging from 53 to 65 years and most of them were HCV infected (85%).

**Table 2:** Epidemiological and clinical characteristics of the patients included in the study.

	Chronic hepatitis	Cirrhosis		HCC	Healthy subjects	p
		Group A	Group B			
No. of patients	17	17	19	26	28	
						
Age (years, mean±SD)	53±13	63±14	65±12	64±12	39±9	<0.0001*
Sex M/F	11/6	12/5	11/8	19/7	17/11	ns
Aetiology						
viral	100%	100%	100%	83%		ns
non viral				17%		

p values according to Kruskall-Wallis ANOVA.

* p = 0.98 (according to Mann-Whitney U test) when patients with cirrhosis and with HCC were compared.

The study was performed according to the principles expressed in the Declaration of Helsinki. Serum samples were collected after obtaining a signed informed consent from the patients, as approved by our institutional Ethics Committee. Serum samples were obtained from whole blood collected into Vacutainer tubes (BD Diagnostics, USA) after centrifugation for 15 min at 2000 ×*g*. Serum was aliquoted into cryovials and stored at −80°C until use.

### ELISA Assay for IgM-linked serpinB3 and serpinB4 Isoforms

Three available monoclonal antibodies (CanAg Diagnostics, Gothenburg Sweden) specific for the two different isoforms of this serpin were utilized: SCC111 antibody, recognizing serpinB3 by Western blot, although a slight cross-reactivity for SerpinB4 in “in solution” assays has been also described recently [37], SCC103 and SCC104 antibodies which reacted only with serpinB4. In particular, the SCC103 monoclonal antibody reacted with serpinB4 complexed with specific proteinase: cathepsinG, while SCC104 did not recognize the serpinB4 complex, but only the free form of the serpin. Polystyrene high binding immunoplates (Sigma Aldrich, Milano, Italy) were coated with 100 µl/well of each monoclonal anti-human serpin antibody diluted in phosphate-buffered saline (PBS) at a concentration of 10 µg/ml and incubated overnight at 4°C. The wells were blocked with 1% bovine serum albumin (BSA)/PBS and incubated at room temperature for 2 h. After washing with PBS-0.05%Tween, 100 µl of serum samples were added at 1∶8 dilution in PBS-0.05%Tween containing 1% BSA and incubated at room temperature for 1 h. The presence of serpinB3-IgM or serpinB4-IgM complexes were revealed by the addition of 100 µl/well of peroxidase-conjugated anti-human IgM (Sigma Aldrich, Milano, Italy) at a 1∶1000 dilution for 1 h. After washing, the enzyme reaction was developed with 100 µl/well of an 2,2′-azinobis(3-ethylbenzthiazoline-6-sulfonic acid-diammonium salt)(ABTS) and hydrogen peroxidase as chromogenic substrates. Plates were analyzed by measuring the optical density at 405 nm on a microtiter plate reader (Tecan, USA). Each sample was tested in duplicate and each run was performed including positive and negative controls. Intra-assay coefficient of variation, calculated by repeated analysis (n = 5) of 3 samples, was 4.6% for serpinB3-IgM assay, 6.2% for serpinB4-IgM (SCC104) assay and 3.8% for serpinB4-IgM (SCC103) assay. The inter-assay coefficients of variation, estimated from five independent runs with 3 samples tested in duplicate, were less than 15% for each assay. Cut-off OD values, calculated as 95^th^percentile on the distribution curve of the specific assay in 28 healthy subjects, were: OD 0.200 for serpinB3-IgM; OD 0.199 for serpinB4-IgM (SCC104Ab) and OD 0.384 for serpinB4-IgM (SCC103Ab). The percentage of positivity for each immunocomplex was determined as the fraction number of patients with immunocomplex levels above the defined cut-off value. The detection limit values (LoD), for each assay, were calculated following the indications of the EP17-A guidelines [38]. LoD was determined by the formula:

LoD = LoB +1.645 (SD _low concentration sample_).

LoB (limit of blank) values were assayed with 10 replicates of the blank reagent (PBS) that contains no analyte. LoD values, calculated with five replicates of a sample known to contain a low concentration of analyte, were OD 0.130 for serpinB3-IgM assay, OD 0.125 for serpinB4-IgM (SCC104Ab) assay, and OD 0.140 for serpinB4-IgM (SCC103Ab) assay. These figures were used for all the calculations when the patients value was below the detection limit.

### ELISA Assay for total SCCA-IgM

Total SCCA-IgM immunocomplexes were also detected in the corresponding serum samples by commercial ELISA Kit (Hepa-IC, generous gift of Xeptagen S.p.A., Venice, Italy) according to manufacturer’s instructions. In this assay a polyclonal anti-human SCCA antibody was used as capture antibody assuring the detection of all SCCA isoforms [21], [39]. The amount of total SCCA-IgM complex was expressed in arbitrary Units/ml (AU/ml). Cut-off value, calculated as 95^th^ percentile, was 156 AU/ml. The assay displayed intra-assay and inter-assay coefficients of variation lower than 10% [21].

### Statistical Analysis

Considering that most variables were not normally distributed, quantitative data were summarized as median and interquartile range (IQR). Comparisons between groups were performed using the non-parametric Mann Whitney U-test and, when more than two groups had to be compared at the same time, the Kruskal-Wallis analysis of variance was performed. Qualitative data were summarized as percentages and the Fisher’s exact test was used for differences in frequencies. A 2 tailed p-value of <0.05 was considered statistically significant. All analyses were performed using GraphPad InStat 3.0 software (San Diego, CA, USA).
